# Significance of C-reactive protein in patients with chronic myelomonocytic leukemia

**DOI:** 10.1007/s10354-022-00981-8

**Published:** 2022-11-28

**Authors:** Jian Liang-Fonseca, Klaus Geissler

**Affiliations:** 1 Medical School, Sigmund Freud University, Vienna, Austria; 2 Department of Internal Medicine V with Hematology, Oncology and Palliative Medicine, Hospital Hietzing, Wolkersbergenstr. 1, 1130 Vienna, Austria

**Keywords:** CMML, C‑reactive protein, Survival, Acute-phase reaction, Glasgow score, Chronische myelomonozytäre Leukämie, C‑reaktives Protein, Überleben, Akute-Phase-Reaktion, Glasgow Score

## Abstract

In a retrospective study, we analyzed the prevalence of elevated C‑reactive protein (CRP) serum levels in 148 patients with chronic myelomonocytic leukemia (CMML), their potential prognostic impact, and potential correlations with laboratory features. Normal, up to 10-fold, and more than 10-fold elevated CRP levels were found in 18%, 59%, and 23% of CMML patients, respectively. Using the CRP cutoff value of 10 mg/L of the widely used Glasgow score, high CRP values were associated with inferior survival (13 vs. 39 months, *p* = 0.014), which retained prognostic significance in multivariate analysis. High CRP values were associated with lower hemoglobin levels. The survival difference between patients with normal (< 5 mg/L) and elevated CRP levels persisted after exclusion of patients with clinical infection. These findings indicate that in CMML patients, the presence of an acute-phase reaction is associated with a poor outcome, independent of clinical infection.

## Abstract (de)

In einer retrospektiven Studie untersuchten die Autoren die Prävalenz erhöhter Werte für C‑reaktives Protein (CRP) bei 148 Patienten mit chronischer myelomonozytärer Leukämie (CMML), ihren potenziellen prognostischen Einfluss und ihre potenzielle Korrelation mit laborchemischen Parametern. Normale, bis 10-fach erhöhte und mehr als 10-fach erhöhte CRP-Werte waren bei 18 %, 59 % bzw. 23 % der CMML-Patienten zu finden. Bei dem CRP-Ccut-off-Wert von 10 mg/l des häufig verwendeten Glasgow Scores waren höhere CRP-Werte mit einem kürzeren Überleben assoziiert (13 vs. 39 Monate, *p* = 0,014), die ihre Signifikanz in der multivariaten Analyse behielten. Hohe CRP-Werte waren mit einem verminderten Hämoglobin vergesellschaftet. Der Überlebensunterschied zwischen Patienten mit normalem und erhöhtem CRP persistierte nach Ausschluss von Patienten mit Symptomen einer Infektion. Diese Ergebnisse zeigen, dass bei CMML-Patienten das Vorliegen einer Akute-Phase-Reaktion mit einem verschlechterten Outcome verbunden ist, unabhängig vom Vorliegen einer klinischen Infektion.

## Introduction

Chronic myelomonocytic leukemia (CMML) is a rare, genotypically and phenotypically heterogenous hematologic malignancy of elderly people, with an intrinsic risk of progression and transformation into secondary acute myeloid leukemia (AML). With regard to the presence of myeloproliferation, CMML was originally subdivided into myeloproliferative disorder (MP-CMML; white blood cell [WBC] count > 13 × 10^9^/L) versus myelodysplastic syndrome (MD-CMML; WBC count ≤ 13 × 10^9^/L MD-CMML) by the FAB criteria [1, 2]. Since CMML is characterized by features of both MDS and MPN, the World Health Organization (WHO) classification of 2002 assigned CMML to the mixed category, MDS/MPN [3]. CMML is further subclassified by WHO into three groups based on blast equivalents (blasts plus promonocytes) in peripheral blood (PB) and bone marrow (BM) as follows: CMML‑0 if PB < 2% and BM < 5% blast equivalents; CMML‑1 if PB 2–4% or BM 5–9% blast equivalents; and CMML‑2 if PB 5–19% or BM 10–19% blast equivalents, and/or Auer rods are present [4]. CMML patients have a highly variable outcome, suggesting that several factors can determine the course of disease and the causes of death in these patients [5–9]. There are a number of established prognostic parameters that have been incorporated into several prognostic models [10–21].

The acute-phase response (APR) is an immediately initiated systemic reaction of the organism to local or systemic disturbances in homeostasis caused by infection, tissue injury, trauma or surgery, neoplastic growth, or immunological disorders [22]. CRP is the most commonly used acute-phase parameter in clinical medicine. The clinical and/or pathophysiological significance of CRP levels in CMML is poorly investigated. Using the database of the Austrian Biodatabase for Chronic Myelomonocytic Leukemia (ABCMML), we analyzed 148 CMML patients with available information on CRP values [23]. This information from a real-life database could be useful in the management of these patients.

## Patients and methods

### Patients

Recently, we have shown that ABCMML may be used as a representative and useful real-life data source for biomedical research [23]. In this database, we retrospectively collected epidemiologic, hematologic, biochemical, clinical, immunophenotypic, cytogenetic, molecular, and biologic data of patients with CMML from different centers. The diagnosis of CMML and leukemic transformation were according to the WHO criteria [2–4]. Clinical and laboratory routine parameters were obtained from patient records. A detailed central manual retrospective chart review was carried out to ensure data quality before analysis of data from institutions. Due to the fact that CMML may be considered as an evolutionary process, from clonal hematopoiesis of indeterminate potential (CHIP) to CMML-related AML [24], and the fact that the distinction between mature and immature monocytic cells, which is required to determine the time of transformation into AML, is notoriously difficult due to the lack of reliable immunophenotypic markers, we found it more appropriate not to exclude the CMML patients with transformation from our analysis [25].

In 148 CMML patients collected between 01.01.1990 and 31.03.2019, information was available regarding CRP values. This research was approved by the ethics committee of the City of Vienna on 10 June 2015 (ethic code: 15-059-VK).

### Statistical analysis

The log-rank test was used to determine whether individual parameters were associated with overall survival (OS). OS was defined as the time from sampling to death (uncensored) or last follow-up (censored). A multivariate Cox regression analysis of overall survival was used to describe the relationship between the event incidence, as expressed by the hazard function, and a set of covariates. Dichotomous variables were compared between different groups using the chi-square test. The Mann–Whitney U test was used to compare two unmatched groups when continuous variables were nonnormally distributed. Results were considered significant at *p* < 0.05. Statistical analyses were performed with SPSS v. 27 (IBM Corp., Armonk, NY, USA); the reported *p*-values are two-sided. A cutoff level of 10 mg/L was taken for CRP, since this value is part of the widely used Glasgow score [26].

## Results

### Patient characteristics

The baseline characteristics of the 148 patients with CMML included in this study are shown in Table 1. In order to make comparisons with other published CMML cohorts possible, the percentages of patients regarding established prognostic parameters are given. As seen in other CMML series, there was a male predominance among study patients and more than half of patients were aged 70 years or older [17]. The proportion of patients with leukocytosis > 13 G/L, anemia < 10 g/dL, thrombocytopenia < 100 G/L, and the presence of blast cells in peripheral blood (PB) was also comparable to other cohorts [17]. Five patients in this cohort had already transformed into CMML-related AML at time of study inclusion.

**Table 1:** Characteristics of chronic myelomonocytic leukemia patients

	Cases (*N* = 148)	Percent
*Age* Evaluable = 148		
< 70 years	46	31
≥ 70 years	102	69
Sex Evaluable = 148		
Male	97	66
Female	51	34
*Leukocytes* Evaluable = 148		
> 13 G/L	76	51
≤ 13 G/L	72	49
*Hemoglobin* Evaluable = 148		
< 10 g/dL	42	32
≥ 10 g/dL	106	68
*Platelets* Evaluable = 148		
< 100 G/L	74	50
≥ 100 G/L	74	40
*Peripheral blood blasts* Evaluable = 126		
Absent	86	68
Present	40	32

### Prevalence of CRP abnormalities in CMML

Normal CRP levels (< 5 mg/L) were found in 27/148 (18%) patients, while 87/148 (59%) patients had a CRP level up to 10-fold higher than the standard (5–50 mg/L), and more than 10-fold elevated CRP levels (> 50 mg/L) appeared in 34/148 (23%) patients with CMML. Taking the CRP cutoff value of 10 mg/L of the widely used Glasgow score, 61/148 (41%) CMML patients had CRP values below, and 87/148 (59%) patients above this level.

### Correlation of increased CRP with laboratory phenotype

As shown in Table 2, CMML patients with CRP values ≥ 10 mg/L had significantly decreased Hb values as compared to CMML patients with CRP levels below this value, whereas other disease features such as leukocyte counts, platelet counts, and circulating blasts were not different.

**Table 2:** Laboratory features stratified by the presence or absence of CRP values ≥ 10 mg/L

	All patients (*N* = 148)	CRP ≥ 10 (*n* = 87)	CRP < 10 (*n* = 61)	*P*-value
Age in years; median (range) Evaluable = 148	75 (36–92)	75 (36–92)	75 (55–90)	0.538
Sex (male); *n* (%) Evaluable = 148	97 (66%)	52 (60%)	45 (74%)	0.083
Leukocytes G/L; median (range) Evaluable = 148	13.1 (3.0–200)	15.4 (3.0–200)	10.8 (3.1–152)	0.177
Hemoglobin g/dL; median (range) Evaluable = 148	11.1 (4.3–16.5)	10.5 (4.3–14.8)	12.2 (5.4–16.5)	0.000
Platelets G/L; median (range) Evaluable = 148	101 (3–718)	102 (5.0–705)	97 (3–718)	0.739
PB blasts %; median (range) Evaluable = 126	0 (0–79)	0 (0–79)	0 (0–38)	0.491

*CRP* C-reactive protein, *PB* peripheral blood

### Impact of increased CRP values on survival

**Fig. 1:**
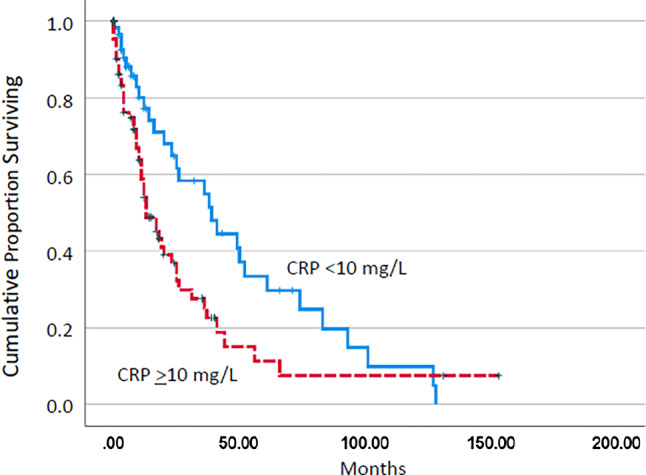
Kaplan–Meier plots for overall survival in chronic myelomonocytic leukemia patients with and without C‑reactive protein (CRP) values ≥ 10 mg/L

The median overall survival (OS) of the three groups (CRP < 5, 5–50, and > 50 mg/L) was 93, 23, and 9 months, respectively (*p* = 0.014). Using the CRP cutoff value 10 mg/L, differences in median OS were 13 vs. 39 months (Fig. 1; *p* = 0.014). Established prognostic parameters including leukocytosis > 13 G/L, thrombocytopenia < 100 G/L, and the presence of blast cells in PB had an adverse impact on survival in univariate analysis (Table 3). There was a borderline association with anemia < 10 g/dL. As shown in Table 4, CRP retained its independent association with OS in multivariate analysis in the presence of other adverse prognostic factors such as leukocytosis, thrombocytopenia, and the presence of circulating blasts, indicating an independent prognostic impact of CRP.

**Table 3:** Univariate analysis of single prognostic parameters in patients with chronic myelomonocytic leukemia

Factors	Factor present Median OS (months)	Factor absent Median OS (months)	*P*-value (log-rank)
CRP > 10 mg/L	13.0	39.0	0.014
WBC > 13 × G/L	16.0	26.0	0.003
Hb < 10 g/dL	11.0	25.0	0.084
PLT < 100 × G/L	11.0	36.0	0.008
PB Blasts present	10.0	25.0	0.006

The log-rank test was used to determine if individual parameters were associated with OS

*CRP* C-reactive protein, *OS* overall survival, *WBC* white blood cell count, *Hb* hemoglobin, *PLT* platelet count, *PB* peripheral blood

**Table 4:** Hazard ratios, confidence intervals, and *p*-values of Cox regression analyses for survival including CRP values ≥ 10 mg/L and prognostic parameters in the univariate analysis

Parameter	Hazard ratio	95% confidence interval	*P*-value
CRP ≥ 10 mg/L	1.711	1.036–2.826	0.036
WBC ≥ 13 G/L	2.235	1.255–3.981	0.006
PLT < 100 G/L	2.190	1.336–3.589	0.002
PB blasts present	1.575	0.939–2.643	0.085

*CRP* C-reactive protein, *WBC* white blood cell count, *PLT* platelet count, *PB* peripheral blood

### Clinical infections

In 46/148 patients, clinical infections were documented. Grade 1, 2, and 3 infections were documented in 26, 18, and 2 patients, respectively. Patients with CRP values ≥ 10 mg/L more often had infections (36/86, 42%) than patients with values < 10 mg/L (10/61, 16%, *p* = 0.001). There was a borderline association to reduced survival in patients with clinical infections grade > 1 (12 vs. 23 months; *p* = 0.087). The significant survival difference between patients with normal and elevated CRP (≥ 5 mg/L) persisted after exclusion of patients with clinical infection (93 vs. 20 months, *p* = 0.043).

## Discussion

Analysis of the acute-phase reaction in CMML may provide some prognostic information which may be useful for patient management but may also give insight into the pathophysiology of disease. CRP has been reported to be a prognostic indicator in a variety of hematologic malignancies [27, 28] and solid tumors [29]. In fact, enhanced CRP is one component of the Glasgow prognostic score, which is a cumulative inflammation-based cancer prognostic marker composed of CRP elevation and a decrease in albumin concentration [26]. In this score, CRP > 10 mg/L and albumin < 35 g/L are used as prognostic factors. Based on this widely used score, we chose 10 mg/L as the cutoff level for CRP in our analysis but did not add albumin, since this value was not regularly available in our real-life cohort. Regarding hematologic diseases, enhanced CRP has been found to have an impact on the clinical outcome in MPN including primary and secondary myelofibrosis, essential thrombocythemia, and polycythemia vera. In a study by Lucijanic et al., higher values of the CRP/albumin ratio (CAR) were able to predict inferior survival in PMF independently of DIPSS (hazard ratio [HR] = 2.17; *p* = 0.015 for high CAR and HR = 2.05; *p* < 0.001 for DIPSS), thus demonstrating its good prognostic potential [28]. In another study by Barbui, a significantly different leukemia-free survival according to hs-CRP levels was documented by Kaplan–Meier analysis [27]. In our study, we could show that CRP is also a prognostic parameter in patients with CMML. The significant survival difference between groups persisted after exclusion of patients with clinical infection. These findings indicate that the presence of an acute-phase reaction is associated with poor outcome, independent of clinical infection.

Recently, inflammation has been demonstrated to act as a major driver in the progression of myeloid malignancies [30]. Regarding BCR/ABL-negative MPN, it has been shown that JAK2 signaling in these diseases leads to chromatin changes that promote NF-κB-induced inflammation and bone marrow fibrosis in MPN models. Most importantly, combined JAK/BET inhibition resulted in a marked reduction in serum levels of inflammatory cytokines, reduced disease burden, and reversed bone marrow fibrosis in vivo. In another preclinical model, a functional link between molecular aberrations and activation of the inflammasome was reported [31]. In this mouse model, Kras-driven myeloproliferation was reversed by functional inactivation of NLRP1, a major component of the inflammasome. A similar phenotypic improvement was seen with therapeutic IL‑1 receptor blockade. Since in our study CRP elevation was also an adverse factor for survival in CMML patients without infection, one is tempted to speculate that inflammation per se may promote progression of this disease. By comparing laboratory parameters of patients with and without CRP elevation, we can see lower hemoglobin values in the high-CRP group, compatible with an inflammatory state in these patients.

We are aware of the limitations of our study. For example, most of the information used in this study was derived from retrospective real-world data that were not collected systematically or prospectively. Thus, not every parameter was available in all patients. In addition, data from patient records were obtained over many years and from many different centers. Moreover, the patients included in this study represented a relatively heterogenous population regarding the blast cell counts. However, real-world data have recently been recognized as an important way to get insights into routine management and the natural history of rare diseases [32]. CMML is a rare disease and adequate patient numbers for a systematic and prospective study are not easy to collect within a limited timeframe. Moreover, the ABCMML provides information derived from molecular as well as from functional studies, and therefore allows a more comprehensive view and deeper insight into the complex pathophysiology of this hematologic malignancy [23].
